# Supplementary material for: Protection of Citrus Fruits from Postharvest Infection with Penicillium digitatum and Degradation of Patulin by Biocontrol Yeast Clavispora lusitaniae 146
Source: Microorganisms. 2020 Sep 25;8(10):1477. doi: 10.3390/microorganisms8101477 (PMC7601000; doi:10.3390/microorganisms8101477)
Supplement: Supplementary file 1 [file microorganisms-08-01477-s001.zip › Supplementary Table S1.pdf]

Table S1: Tabulated values of χ2 statistic according to Amerine et al. [53].

	5%	1%	0.1%
One-sided (difference)	2.71	5.41	9.55
Two-sided (preference)	3.84	6.64	10.83

Formula used for χ2 test:

$$\chi_c^2 = \sum \frac{(O_i - E_i)^2}{E_i}$$

Reference

53. Amerine, M.A.; Pangborn, R.M; Roessler, E.B. Statistical Procedures. In *Principles of sensory evaluation of food*; Amerine, M.A.; Pangborn, R.M; Roessler, E.B.; Elsevier: Netherlands, 1965; pp. 437–493, doi: 10.1016/C2013-0-08103-0.
